# Supplementary material for: Predicting Body Weight from Birth to Old Age in Giant Pandas Using Machine Learning
Source: Animals (Basel). 2024 Dec 20;14(24):3694. doi: 10.3390/ani14243694 (PMC11672812; doi:10.3390/ani14243694)

Supplementary Materials

Supplementary Table S1. Initial hyperparameters of machine learning algorithms

Algorithm	Library	Hyperparameters
KMeans	scikit-learn (0.19.2)	n_clusters=2, init='k-means++', n_init=10, max_iter=300, tol=0.0001, precompute_distances='auto', copy_x=True, algorithm='auto'
RandomForestRegressor	scikit-learn (0.19.2)	bootstrap=True, criterion='mse', max_depth=None, max_features='auto', max_leaf_nodes=None, min_impurity_decrease=0.0, min_samples_leaf=1, min_samples_split=2, min_weight_fraction_leaf=0.0, n_estimators=100, oob_score=False, warm_start=False
ExtraTreesRegressor	scikit-learn (0.19.2)	bootstrap=False, criterion='mse', max_depth=None, max_features='auto', max_leaf_nodes=None, min_impurity_decrease=0.0, min_samples_leaf=1, min_samples_split=2, min_weight_fraction_leaf=0.0, n_estimators=100, oob_score=False, warm_start=False
AdaBoostRegressor	scikit-learn (0.19.2)	base_estimator=None, learning_rate=1.0, loss='linear', n_estimators=100
GradientBoostingRegressor	scikit-learn (0.19.2)	alpha=0.9, criterion='friedman_mse', init=None, learning_rate=0.1, loss='ls', max_depth=3, max_features=None, max_leaf_nodes=None, min_impurity_decrease=0.0, min_samples_leaf=1, min_samples_split=2, min_weight_fraction_leaf=0.0, n_estimators=100, presort='auto', subsample=1.0, warm_start=False
XGBRegressor	xgboost (1.5.1)	n_estimators=100, objective='reg:squarederror'

The initial hyperparameters of the k-means, Random Forests, Extremely Randomized Trees, AdaBoost, Gradient Tree Boosting and XGBoost methods in Python (3.7.0) are listed in Table S1.

Supplementary Table S2 Weight-for-age (in days) chart of giant pandas from birth to old age

A: Female; B: Male. Owing to size constraints, Table S2 is presented as a separate supplement.

Supplementary Figure S1. Distribution of predicted weight vs actual weight on the test set of sub-adults/adults without calibration of weighing on an empty stomach

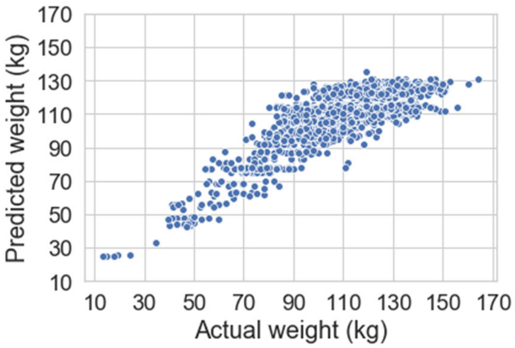

R^2 score = 0.684, MAE = 8.8 kg, MSE = 126.8 kg^2.

Before calibrating weighing on an empty stomach, a baseline model was trained on the training set from the initial data set of sub-adults/adults refer to Figure 1C. The first evaluation results are shown in Table S2. The R^2 scores were 0.461~0.660, indicating poor performance. The train_R^2 mean values of the RF, ET and XGB models were much larger than their R^2 mean values, indicating that these models showed overfitting. The GBDT model was used for hyperparameter optimization, but the results were not significantly improved. Therefore, the initial hyperparameters were retained. The second evaluation was performed on the test set (Figure 1C), which resulted in an R^2 score of 0.684, a MAE of 8.8 kg and a MSE of 126.8 kg^2. The distribution of the predicted weight and actual weight is shown in Figure S1, from which the points do not converge on the diagonal line. These evaluation results indicate that the prediction ability of the model needs to be improved.

Supplementary Figure S2. Fit curves and equations of weight vs age (in days) for the sub-adult pandas

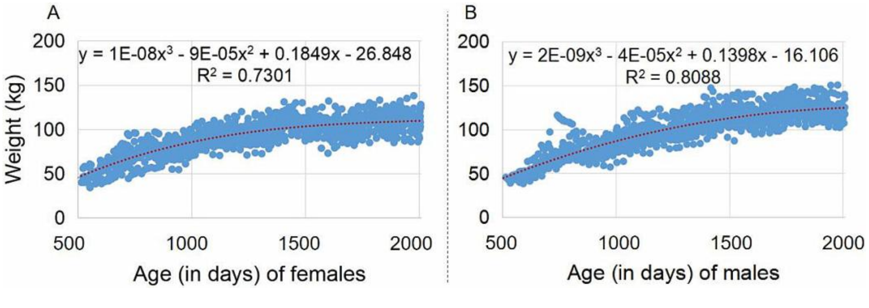

A: Fit curve and equation of the females. B: Fit curve and equation of the males.

Supplementary Figure S3. Distribution of the predicted weight vs actual weight on the test set

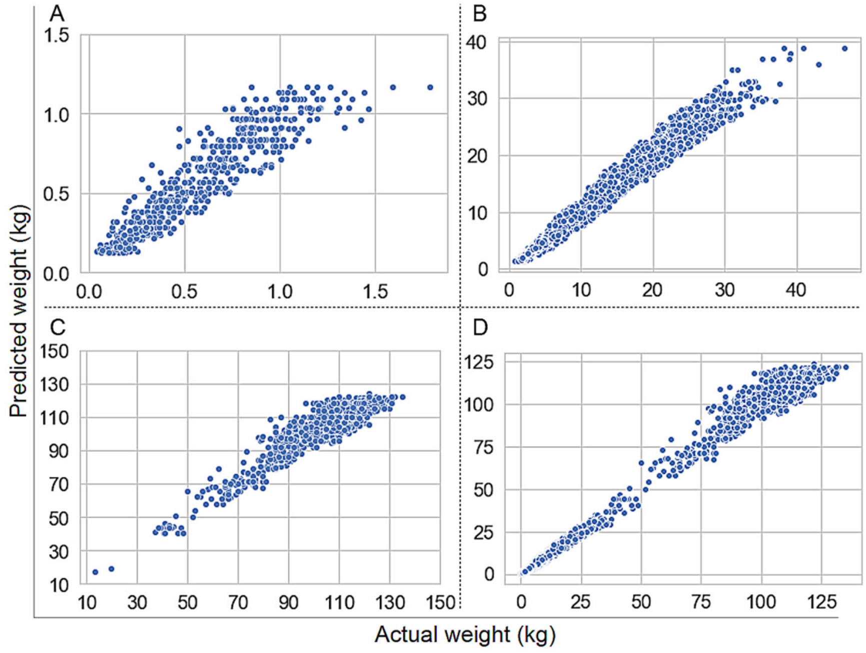

A: Prediction for cubs of 0~30 days of age. R2 score = 0.883, MAE = 0.081 kg and MSE = 0.013 kg2. B: Prediction for cubs of 31~500 days of age. R2 score = 0.973, MAE = 0.895 kg, MSE = 1.618 kg2. C: Prediction for sub-adults/adults (over 501 days of age). R2 score = 0.850, MAE = 5.2 kg and MSE = 42.7 kg2. D: Distribution obtained by merging the three aforementioned predictions.

Supplementary methods: An example for adjusting the weights of sub-adult pandas by month

1) Fitted curve and equation for weight and age of sub-adult male pandas.

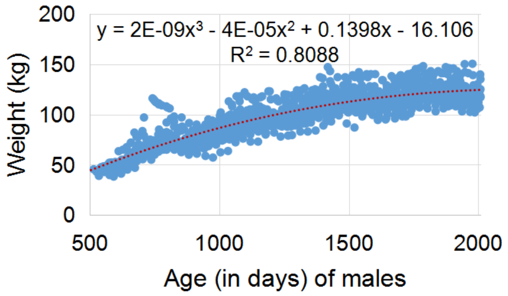

2) An example of recorded body weights of sub-adult male pandas aged 31 months of age.

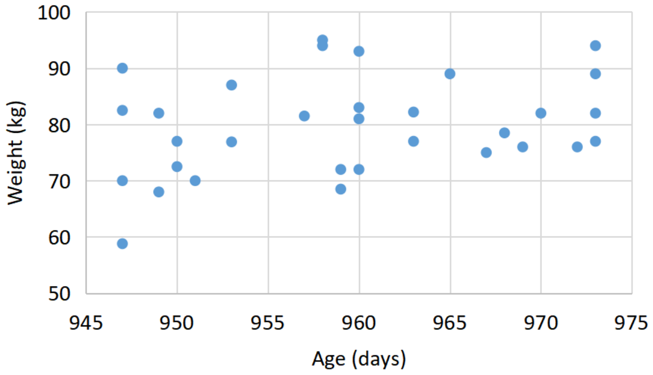

3) Calculate the mean day (*MD*) of record days (days with weight recorded) in the month.

$$MD = \frac{947\times4+949\times2+950\times2+951+953\times2+957+958\times2+959\times2+960\times4+963\times2+965+967+968+969+970+972+973\times4}{4+2+2+1+2+1+2+2+4+2+1+1+1+1+1+1+4} = 959.5$$

4) Calculate the body weight (*W*) in the mean day by the fitted equation.

$$W = 2\times10^{-9}\times MD^3 - 4\times10^{-5}\times MD^2 + 0.1398\times MD - 16.106 = 83.0\,\text{kg}$$

5) Adjust each weight value in the day with weight recorded.

Age (days)	Weight (kg)	Weight calculated by fitted equation (*WC**, kg)	Difference of weight between mean day and each record day on fitted curve (*D***, kg)	Calculated weight in the mean day (*WM****, kg)
947	90	82.1	0.9	90.9
947	70	82.1	0.9	70.9
947	82.5	82.1	0.9	83.4
947	58.8	82.1	0.9	59.7
949	82	82.2	0.8	82.8
949	68	82.2	0.8	68.8
950	72.5	82.3	0.7	73.2
950	77	82.3	0.7	77.7
951	70	82.4	0.6	70.6
953	87	82.5	0.5	87.5
953	76.9	82.5	0.5	77.4
957	81.5	82.8	0.2	81.7
958	94	82.9	0.1	94.1
958	95	82.9	0.1	95.1
959	72	82.9	0.1	72.1
959	68.5	82.9	0.1	68.6
960	72	83	0	72
960	83	83	0	83
960	81	83	0	81
960	93	83	0	93

963	77	83.2	-0.2	76.8
963	82.2	83.2	-0.2	82
965	89	83.3	-0.3	88.7
967	75	83.5	-0.5	74.5
968	78.5	83.6	-0.6	77.9
969	76	83.6	-0.6	75.4
970	82	83.7	-0.7	81.3
972	76	83.8	-0.8	75.2
973	77	83.9	-0.9	76.1
973	82	83.9	-0.9	81.1
973	94	83.9	-0.9	93.1
973	89	83.9	-0.9	88.1

$* \, WC = 2 \times 10^{-9} \times Age^3 - 4 \times 10^{-5} \times Age^2 + 0.1398 \times Age - 16.106$

$** \, D = W - WC$

$*** \, WM = Weight + D$

6) Calculated weight in the mean day

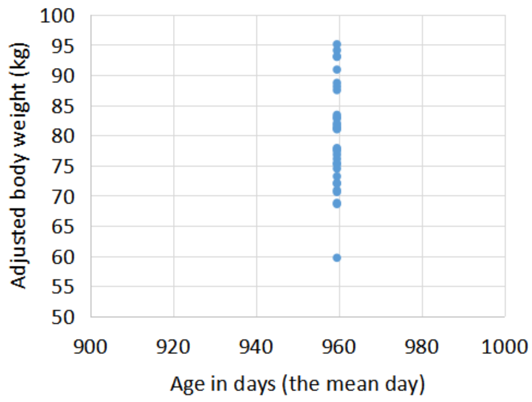

Supplement: Supplementary file 1 [file animals-14-03694-s001.zip › animals-3338507-supplementary.pdf]
